# Anticholinergic syndrome due to mydriatic drops intoxication in a child: A case report

**DOI:** 10.1016/j.toxrep.2024.101889

**Published:** 2025-01-07

**Authors:** J. Serralabós-Ferré, I. Barceló-Carceller

**Affiliations:** Pediatrics, Hospital de la Santa Creu i Sant Pau, Barcelona, Spain

**Keywords:** Anticholinergic syndrome, Mydriatic drops, Cyclopentolate intoxication, Case report

## Abstract

Anticholinergic syndrome consists of neurological and systemic symptoms, including agitation, mydriasis and dryness that can be an adverse reaction to a number of medications, some of them as seemly harmless as cycloplegic mydriatic drops. We present the case of a 7-year-old previously healthy female child who presented to the emergency room with impaired neurological status, inability to recognize family members and incoherent speech, as well as facial flushing, mucosal dryness and bilateral mydriasis after having mistakenly received a high dose of mydriatic drops. She made a complete recovery without need for treatment and had no sequelae. It has been described that anticholinergic syndrome can be related to a number of mydriatic drops (such as cyclopentolate) as well as other medications, in adults, children and neonates. It is a clinical diagnosis and requires no etiological testing. Depending on the severity at presentation, it may be required to administer the antidote physostigmine.

## Highlights

• Anticholinergic syndrome can manifest with dryness, facial flushing and neurological symptoms ranging from confusion to delirium and hallucinations.
• Anticholinergic syndrome should be considered as a potential adverse effect of various drugs, including mydriatic drops.
• It is important to prescribe and communicate with parents well when using apparently harmless medications.
• Pediatric intoxications are rare and should be reported to extend and improve scientific knowledge.

## Graphical Abstract

**Figure:**
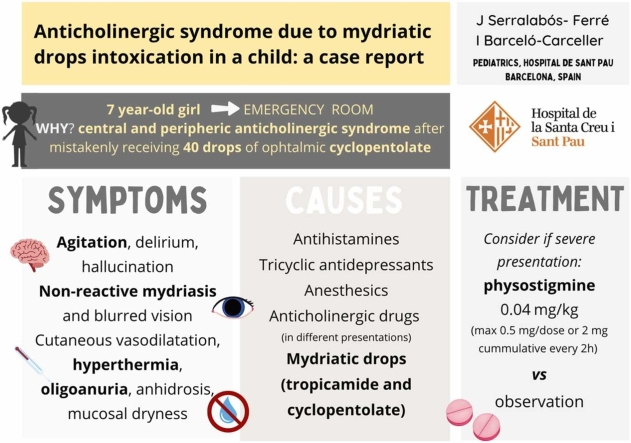

## 1 Introduction

Anticholinergic syndrome consists of a series of symptoms resulting from the blockade of cholinergic receptors. The classic clinical presentation includes agitation, delirium and hallucinations, non-reactive mydriasis and blurred vision, cutaneous vasodilation and hyperthermia, anhidrosis and mucosal dryness, as well as oligoanuria. Toxicity can be classified as central or peripheral based on the predominant effect on the nervous system, with central manifestations being considered a form of “severe” toxicity [1].

Anticholinergic adverse reactions presenting as anticholinergic syndrome have been described in association with many commonly prescribed medications, including antihistamines, tricyclic antidepressants, anaesthesics and both intravenous and topical anticholinergic agents. Pupil dilation is a routine procedure in ophthalmology for diagnostic and therapeutic purposes, often using anticholinergic agents such as tropicamide and cyclopentolate. Although these are generally safe medications, their repeated or inadequate administration may lead to toxicity, particularly in vulnerable populations such as children [1]. Despite that, the published literature about paediatric population is scarce.

We present a case report of a paediatric patient who developed an anticholinergic syndrome after the administration of cyclopentolate mydriatic drops. We aim to highlight the importance of caution when prescribing and administering mydriatic drops, especially in children. Through this report, we aim to contribute to the existing literature on pediatric toxicology and ophthalmologic emergencies, and to raise awareness of anticholinergic syndrome associated with mydriatic drops.

## 2 Case presentation

A 7-year-old girl with no previous medical history presented to the paediatric emergency department accompanied by her mother, reporting abnormal behaviour over the previous 3 hours, including failure to recognise family members, incoherent speech and dizziness. She presented with facial and hand flushing, mouth dryness and labial oedema which had started earlier that day, 30 minutes after she had accidentally received 20 drops of cyclopentolate in each eye in preparation for a routine ophthalmology evaluation. The administration had been accidental due to a misunderstanding between the mother and the healthcare professional. These 20 drops had been applied only once over the course of 2 minutes.

**Fig. 1:**
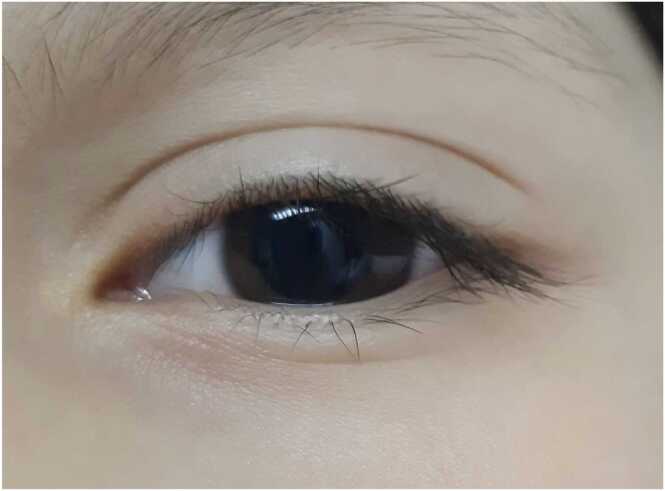
Mydriatic eye.

Physical examination revealed the aforementioned findings, as well as bilateral non-reactive mydriasis (Fig. 1), altered speech, and an otherwise normal neurological examination. Her vital signs were stable with a normal electrocardiogram. No further diagnostic testing was performed. The patient’s symptoms improved gradually until complete resolution without treatment. She was kept under observation for 4 hours and normal vital signs, neurological status and present diuresis were confirmed prior to discharge.

The symptoms and clinical history were consistent with a case of central and peripheral anticholinergic syndrome due to mydriatic drop toxicity. She did not experience similar episodes thereafter.

## 3 Discussion

The diagnosis of anticholinergic syndrome should be considered in children presenting with inconsistent neurological symptoms, and patients should be questioned about potential drug exposure during medical evaluation. Although cycloplegic agents are generally considered to be very safe medications, there are rare adverse reactions associated with their use. Existing literature highlights varying degrees of adverse reactions (from flushing to delirium), particularly in relation to atropine and cyclopentolate drops. Tropicamide is often regarded as safer, with several studies reporting an absence of adverse reactions [2].

On the other hand, results regarding atropine and cyclopentolate are more varied. Some studies report adverse reactions in up to 24 % of patients [3] after repeated instillation, while others show no severe effects apart from drowsiness in a small percentage of subjects [4] or even no side effects [5]. Data on serious side effects is rare, most of it coming from case reports, including cases of hallucination and agitation in adults [6], [7], as well as paediatric cases such as a 4-year-old girl experiencing drowsiness, agitation, and hallucinations after receiving 24 drops of cyclopentolate [8].

Neonates being screened for retinopathy of prematurity may experience adverse effects more frequently, with cases reported of flushing, hyperthermia and agitation following excessive administration of cyclopentolate [9] and even two neonates who suffered transient paralytic ileus after cyclopentolate-phenylephrine eye drops [10]. Additionally, a fatal case of necrotising enterocolitis associated with cyclopentolate use has been documented [11].

There are also reports in the literature of anticholinergic syndrome associated with other medications, such as both general and regional anaesthetics [12], hyoscine hydrobromide [13], antihistamines [14], or home remedies containing *Atropa belladonna* [15].

The mechanism explaining the anticholinergic syndrome as a side effect of mydriatic drops includes antagonism of acetylcholine receptors in the postganglionic fibers of the parasympathetic nervous system, and can present at first as excitatory and secondly as inhibitory in the central nervous system [7]. The effect appears to be dose-related, with neonates and children being especially susceptible, particularly those with low weight [4]; therefore it is advised to avoid repeated instillations. It is crucial to provide clear instructions for drug administration, even for topical agents, which can be systemically absorbed at rates of 30–80 % through the conjunctiva and nasopharyngeal mucosa. Some authors describe the possibility of idyosincratic reaction to atropine eye drops, such as a case when therapeutic dose of atropine was administered [16]. The dose administered in our case was well above the limit recommended by our national guidelines (1 drop of cyclopentolate for children over 6 years) [17]. This fact, combined with previous reports indicating that the most common effect is dose-related [4], suggests that our patient’s symptoms were likely due to an inadvertent overdose of mydriatic drops.

Treatment may be required in some cases, particularly those with severe central or peripheral anticholinergic syndrome. The antidote physostigmine is generally recommended at a dose of 0.04 mg/kg (maximum 0,5 mg/dose initially or cumulatively up to 2 mg in repeated doses) either intravenously or intramuscularly. Some patients may require successive doses after approximately 2 hours due to its pharmacological properties (half-life elimination 1–2 hours) [18].

## 4 Conclusion

Anticholinergic syndrome may present with dryness, facial flushing and neurological symptoms ranging from confusion to delirium and hallucinations. This syndrome should be considered as a potential adverse effect of various drugs, including mydriatic drops. Thus, checking for recommended doses, correct and clear prescription and communication with parents or caregivers are essential even with apparently harmless medications. Paediatric intoxications with anticholinergic agents are rare but should be reported to extend scientific knowledge and to improve paediatric patient care.

## CRediT authorship contribution statement

**J. Serralabós-Ferré:** Conceptualization, Data curation, Investigation, Visualization, Writing – original draft. **I. Barceló-Carceller:** Conceptualization, Supervision, and Writing corrections and editing.

## Declaration of Competing Interest

The authors declare that they have no known competing financial interests or personal relationships that could have appeared to influence the work reported in this paper.

## Data Availability

The data that has been used is confidential.
